# Characteristics and related factors of nonfatal injuries among adolescents and college students in Shenzhen city of China

**DOI:** 10.1186/1471-2458-13-392

**Published:** 2013-04-26

**Authors:** Li Zhou, Dingyan Chen, Guoying Dong

**Affiliations:** 1 Shenzhen Center for Disease Control and Prevention, No. 8 Longyuan Road, Nanshan District, Shenzhen, Guangdong Province 518055, China

**Keywords:** Injury risk, Injury rate, Adolescent, Students

## Abstract

**Background:**

Injuries impact adolescents and young adults in unique ways. The purpose of this study was to determine the incidence rate of nonfatal injuries, and identify characteristics and risk factors for the injuries among adolescents and college students in Shenzhen, China.

**Methods:**

A total of 4,138 students from 79 classes were selected using a purposive sampling method in 2010. The questionnaire included personal demographics, behavioral factors, and self-perceived agrypnia. Stepwise multivariate logistic regression models were used to explore the risk factors of injury.

**Results:**

The annual incidence rate of nonfatal injuries was 13.5%. Injuries were significantly correlated with gender (boys vs. girls, adjusted odds ratio [OR], 1.58, 95% confidence interval [CI], 1.30-1.93) and self-perceived agrypnia (sometimes vs. no, adjusted OR, 1.64, 95% CI, 1.31-2.05; often vs. no, adjusted OR, 2.34, 95% CI, 1.74-3.14), attending PE class ( >2 classes/week vs. ≤ 2 classes/week, adjusted OR, 1.25, 95% CI, 1.04-1.51), sexual behaviors (yes vs. no, adjusted OR, 1.46, 95% CI, 1.03-2.07), physical fighting (yes vs. no, adjusted OR, 1.84, 95% CI, 1.49-2.28), alcohol consumption (yes vs. no, adjusted OR, 1.29, 95% CI, 1.06-1.59), unsafe cycling (yes vs. no, adjusted OR, 1.47, 95% CI, 1.20-1.80) and skating in unsafe places (yes vs. no, adjusted OR, 1.57, 95% CI, 1.10-2.24). Additionally, falls were the leading cause of injuries, and gymnasiums of schools were the most-reported places where injuries occurred.

**Conclusions:**

Nonfatal injuries have turned into a pressing public health problem among adolescents and college students in Shenzhen, China. Strategies targeting the risk factors may be effective for the prevention of injuries.

## Background

Injuries are an emerging public health problem around the world that affect all age population, especially adolescents and young adults. It is reported that globally an estimated 5.8 million people die from injuries each year [1]. For people aged 5 to 29 years old, injuries are the leading cause of death [1]. In particular, injuries and injury-related deaths have an inestimable impact on the families and communities affected, whose lives are often irrevocably altered by the tragedies. In China, more than 10% of all deaths result from injuries and premature mortality, accounting for more than 30% of all potentially productive years of life lost [2]. The Ministry of Health of China reported that the annual incidence of injuries for all-age population was between 16.1% and 21.9%, and 700–750,000 people die as a result of injuries each year in China [3,4]. Additionally, injuries incidence among youths was reported to be about 50% [5]. As a result, it is desirable to conduct prevention research to protect young people against injuries. A precondition for developing effective prevention strategies to cope with the rapid growth of injuries is a sufficient understanding of the incidence and risk factors of injuries. In the past several decades, many studies have been conducted on injuries among the young population [6-11]. However, most published studies that identified various socio-demographic and behavioral factors related to injuries have focused on the populations in developed countries, and the information in developing countries remains unclear.

Shenzhen, located in the southern tip of China and neighboring Hong Kong, was the first Special Economic Zone established in China in the 1980s. The educational system in Shenzhen has developed rapidly as a consequence of rapid economic development, resulting in large numbers of students and schools spread throughout the city. Since injury is particularly likely to affect students, in this study we aimed to find out (1) What was the incidence rate of nonfatal injuries among adolescents and college students in Shenzhen? (2) What were the characteristics of these injuries? (3) What were the major risk factors among adolescents and the college students? The goal was to develop suggestions for more effective measures in injuries prevention in the future.

## Methods

### Study area, subjects and sampling method

Data for this study were collected using a cross-sectional survey design. The sample pool included all the districts in Shenzhen City. According to the Shenzhen Municipal Bureau of Statistics, there were 10.35 million permanent residents living in the city in 2010 [12], making Shenzhen one of the largest cities in China. Additionally, the educational system has also developed rapidly. By the end of 2009, there were 1,636 schools and around 1.27 million students in Shenzhen [13]. We utilized a purposive sampling method to select 16 schools (7 junior high schools, 7 senior high schools and 2 colleges) from 6 districts of Shenzhen. We used grade as stratum, selecting classes by cluster sampling in each stratum. All the students in the selected classes agreeing to participate in the survey were recruited. In total, 4,209 students in 79 selected classes were surveyed and 4,138 of them completed the survey correctly, yielding a response rate of 97.3%.

### Data collection procedure

The data were collected between October 2010 and December 2010 in Shenzhen using the questionnaire of the Guangdong Provincial Children’s Health Behavior Survey (GPCHBS), which was designed by the Children and Adolescents/School Health Center of the Chinese Center for Disease Control and Prevention. The GPCHBS is cross-sectionally administered in 21 areas (8 urban and 13 rural areas) in Guangdong province every three years. Shenzhen is one of the selected areas. The questionnaire was administered voluntarily and anonymity was assured to protect the respondents’ privacy. All investigators were trained before the survey.

The dependent variable was measured by the response to a question: “During the past 12 months, how many times were you seriously injured?” A serious injury was defined as an injury that required treatment by a doctor or nurse or resulted in missing at least one full day of usual activities (such as going to school or taking part in sports).” The variable was dichotomized into “No” vs. “Yes” for all the analyses. Two follow-up questions were asked about symptoms of the injuries and places where injuries occurred, respectively.

Independent variables consisted of demographic characteristics (including gender, age, height, weight, grade, registered permanent residence), behavioral factors (trying smoking, alcohol consumption, unsafe cycling, not walking on zebra crossing lines or pedestrian overpass or underpass, swimming in unsafe places, skating in unsafe places, physical fighting, sexual behaviors, attending physical education (PE) classes), and self-perceived insomnia. Body mass index (BMI) was calculated as weight (kg) divided by the square of height (m^2^). Age- and sex-specific BMI cut-off points developed by the Working Group for Obesity Task Force in China was used to identify overweight and obesity. Overweight was defined as BMI between the 85th and 95th percentile, obesity was defined as BMI at the 95th percentile or higher, and normal weight was defined as BMI lower than 85th percentile [14,15]. To assess unsafe cycling, six questions were asked, including “did you ever ride a bike with both hands off the handlebars”, “did you ever ride a bike with one or both hand(s) clinging to other vehicles”, “did you ever chase with each other when riding a bike”, “did you ever ride a bike against the traffic flow”, “did you ever carry persons when riding a bike”, and “did you ever ride a bike ignoring the traffic-control signals”. A student was considered to have unsafe cycling behavior if answering “yes” to any one of the six questions.

### Statistical analysis

The incidence rate of injuries was estimated and compared in relation to the demographics, behavioral factors, and self-perceived agrypnia. χ^2^ test was used to test the differences in injury incidence among different groups. Multivariate logistic regression analyses were performed to determine the risk factors for students’ injury. All the independent variables, including gender, registered permanent residence, BMI category, grade, self-perceived agrypnia, classes of PE, sexual behavior, trying smoking, alcohol consumption, physical fighting, unsafe cycling, skating in unsafe places, walking on the zebra crossing or pedestrian overpass or underpass, swimming in unsafe places, were step-wisely selected with criteria of *P*<0.05 for entry and *P*<0.10 for removal. Odds Ratios (OR) and 95% confidence interval (CI) were computed to assess the associations between the risk factors and injuries. All the p-values presented were two-sided with a significance level of 0.05. All the analyses were carried out using the software package SPSS for Windows (Version 12.0).

The study was approved by the Ethics Committee of Shenzhen Centre for Disease Control and Prevention.

## Results

### Demographics of study sample

The final study sample consisted of 4,138 students, including 2,134 boys and 2,004 girls. Among them, there were 2,302 junior high school students, 1,138 senior high school students and 698 college students. The average age was 15.3 years old, ranging from 10 to 24 years old.

### Injury incidence

A total of 560 (13.5%) students reported to have at least one injury during the past 12 months. Table 1 presents the frequency and the annual incidence rate of injuries by demographic characteristics, behavioral factors, self-perceived agrypnia, and PE courses. Male students had significantly higher injury incidence as compared with the female students (17.10% vs. 9.73%, *P*<0.001). Additionally, crude injury incidence was significantly higher among the students who attended PE class >2 classes/week (15.30%), and often had self-perceived agrypnia (22.75%). Crude injury incidence was also significantly higher for the students who had the following behaviors: sexual behaviors (23.83%), trying smoking (17.11%), alcohol consumption (15.23%), physical fighting (24.68%), unsafe cycling (20.20%), skating in unsafe places (25.38%), swimming in unsafe places (21.70%), and not walking on the zebra crossing lines or pedestrian overpass or underpass (15.84%).

**Table 1:** Injury incidence and the relationship between the demographic and behavior characteristics and injuries in a sample of 4,138 students in Shenzhen, China

**Variables**	**N**	**Injury**		**Non-injury**		***χ***^***2***^	***P***
		**n**	**%**	**n**	**%**		
Gender							
Male	2,134	365	17.10	1,769	82.90	48.02	<0.001
Female	2,004	195	9.73	1,809	90.27		
Registered permanent residence							
Local	3,026	414	13.68	2,612	86.32	0.21	0.645
Non-local	1,112	146	13.13	966	86.87		
BMI Category							
Normal weight	3,727	492	13.20	3,235	86.80	3.96	0.138
Overweight	308	49	15.91	259	84.09		
Obesity	103	19	18.45	84	81.55		
Grade							
Primary school	2,302	327	14.21	1,975	85.79	2.62	0.270
Secondary school	1,138	150	13.18	988	86.82		
College	698	83	11.89	615	88.11		
Self-perceived agrypnia							
No	1,412	130	9.21	1,282	90.79	57.64	<0.001
Sometimes	2,260	324	14.34	1,936	85.66		
Often	466	106	22.75	360	77.25		
Having PE class							
≤2 classes/week	2,184	261	11.95	1,923	88.05	9.90	0.002
>2 classes/week	1,954	299	15.30	1,655	84.70		
Sexual behavior							
No	3,924	509	12.97	3,415	87.03	20.45	<0.001
Yes	214	51	23.83	163	76.17		
Trying smoking							
No	3,074	378	12.30	2,696	87.70	15.62	<0.001
Yes	1,064	182	17.11	882	82.89		
Alcohol consumption							
No	1,505	159	10.56	1,346	89.44	17.81	<0.001
Yes	2,633	401	15.23	2,232	84.77		
Physical fighting							
No	3,360	368	10.95	2,992	89.05	101.72	<0.001
Yes	778	192	24.68	586	75.32		
Unsafe cycling							
No	3,123	355	11.37	2,768	88.63	51.04	<0.001
Yes	1,015	205	20.20	810	79.80		
Skating in unsafe places							
No	3,941	510	12.94	3,431	87.06	24.81	<0.001
Yes	197	50	25.38	147	74.62		
Walking on the zebra crossing or pedestrian overpass or underpass							
No	1,976	313	15.84	1,663	84.16	17.20	<0.001
Yes	2,162	247	11.42	1,915	88.58		
Swimming in unsafe places							
No	3,820	491	12.85	3,329	87.15	19.63	<0.001
Yes	318	69	21.70	249	78.30		

BMI, body mass index; PE, physical education.

### Risk factors for injuries

Table 2 shows the unadjusted and adjusted ORs and their 95% CIs of risk factors for injuries. Results from stepwise multivariate logistic regression analyses revealed that the students with the following risk factors were at significantly higher risk of injuries: male gender (adjusted OR, 1.58, 95% CI, 1.30-1.93), self-perceived agrypnia (sometimes vs. no, adjusted OR, 164, 95% CI, 1.31-2.05; often vs. no, adjusted OR, 2.34, 95% CI, 1.74-3.14), attending PE class >2 classes/week (adjusted OR, 1.25, 95% CI, 1.04-1.51), sexual behaviors (adjusted OR, 1.46, 95% CI, 1.03-2.07), physical fighting (adjusted OR, 1.84, 95% CI, 1.49-2.28), alcohol consumption (adjusted OR, 1.29, 95% CI, 1.06-1.59), unsafe cycling (adjusted OR, 1.47, 95% CI, 1.20-1.80), and skating in unsafe places (adjusted OR, 1.57, 95% CI, 1.10-2.24).

**Table 2:** Unadjusted and adjusted associations between the predictor variables and injuries in a sample of 4,138 students in Shenzhen, China

**Variables**	**Crude OR (95% CI)**	**Adjusted OR (95% CI)**
Gender		
Male	1.91 (1.59-2.30)	1.58 (1.30-1.93)
Female	1.00	1.00
Self-perceived agrypnia		
None	1.00	1.00
Sometimes	1.65 (1.33-2.05)	1.64 (1.31-2.05)
Often	2.9 (2.19-3.85)	2.34 (1.74-3.14)
Having PE class		
≤2 classes/week	1.00	1.00
>2 classes/week	1.33 (1.11-1.59)	1.25 (1.04-1.51)
Sexual behavior		
No	1.00	1.00
Yes	2.10 (1.51-2.91)	1.46 (1.03-2.07)
Alcohol consumption		
No	1.00	1.00
Yes	1.52 (1.25-1.85)	1.29 (1.06-1.59)
Physical fighting		
No	1.00	1.00
Yes	2.66 (2.19-3.24)	1.84 (1.49-2.28)
Unsafe cycling		
No	1.00	1.00
Yes	1.97 (1.63-2.38)	1.47 (1.20-1.80)
Skating in unsafe places		
No	1.00	1.00
Yes	2.29 (1.64-3.20)	1.57 (1.10-2.24)

OR, odds ratio; CI, confidence interval; PE, physical education.

Adjusted for all variables in Table 1.

### Characteristics of injuries

Among the 560 students injured, the leading cause of the injuries was falls. Of them, 170 (30.4%) students reported having had falls, 113 (20.2%) students reported being injured with hard objects, 87 (15.5%) students had cuts or piercings, 33 (5.9%) students had animal bites, and 47 (8.4%) students were injured by other causes, including explosion, electrocution, suffocation, drowning and poisoning. Additionally, 119 (21.3%) students had injuries in gymnasiums of schools, 71 (12.7%) students were injured at home and 50 (8.9%) students had injuries in the schools but not gymnasiums. The remaining injuries occurred in other places, including roads, stations, supermarkets, and so on.

## Discussion

In the current study, we investigated the incidence rate, associated risk factors and characteristics of injuries among adolescents and young adults in Shenzhen city, Guangdong province, China. We found that close to a quarter of the subjects experienced at least one injury in the past 12 months, which was consistent with that of undergraduates in Wenzhou city, Zhejiang province, China as reported by Shi et al. (13.99%) [3]. However, the incidence rate was higher than another study in Shandong province, China (6.67%) [16].

We found that male students had a higher rate of injuries as compared with the female students, which was in line with the results from previous studies [17-20]. This is probably because male students have higher levels of sensation seeking [21-23] and are more impulsive [20] thus are more likely to engage in risky behavior than the female students [20,24]. We also found that self-perceived agrypnia was associated with students’ injuries. Similarly, a previous study in Guangxi province also suggested that among adolescents, a short duration of sleep could be a potential risk factor for unintentional injuries [25]. Sleepiness and fatigue have been shown to be major risk factors for injury. Therefore, self-perceived agrypnia may lead to sleeping in the daytime, therefore increasing the risk of injuries. Hence, early identification and early treatment of sleep problems including self-perceived agrypnia may have great significance in preventing potential injuries and the consequences. Moreover, students’ behaviors, such as sexual behaviors, physical fighting, and alcohol consumption also contributed to injuries. These findings are also consistent with previous studies. In a study of adolescents in the United States reported by Hammig et al. [6], fighting was significantly associated with higher incidence of injuries. Pickett et al. [26] reported that drunkenness was associated with medically treated, street, and fighting injuries among adolescents. Dong et al., [17] reported that risk behavior index could increase the risk of road traffic injury. It was also reported that risk taking and sensation seeking were significantly associated with sexual experience, alcohol use and physical fighting [27]. Additionally, children and adolescents’ temperament is a risk factor for increased rates of injury. Increasing levels of independence may coincide with more challenging behaviors in this population. The “risk taking” characteristic may determine their behaviors like alcohol use and physical fighting, which contribute to injuries. Therefore, we suggest that an early understanding of students’ characteristics and their behaviors could provide opportunities for early intervention and thus prevent potential injuries and related consequences.

Additionally, unsafe cycling is a dangerous behavior that leads to injuries. For example in the United States, reports show that collisions with motor vehicles resulted in 48,000 police-reported nonfatal injuries per year among cyclists and cyclists had the second highest nonfatal traffic injury rate [28]. Thus, measures that prevent injuries for bicyclists are needed given the benefits of physical activity that come with active travel and more attention needs to be paid to this activity. Preventing unsafe cycling requires the united efforts of individuals and the managements. Strategies focusing on specific forms of behavior like helmet wearing [29], using lighting for bicycles, forbidding riding against the traffic flow and punishment for unsafe cycling may be effective in this kind of injury prevention.

It should be noted that in the univariate analyses of the current study, having sexual behavior was a higher risk factor for injury than unsafe cycling. The following two reasons may partly explain that: firstly, the two factors may be affected by each other or by other confounders, because when we conducted multivariate logistic regression analyses, the ORs of sexual behaviors and unsafe cycling were very close; secondly, sexual behavior may reflect an aspect of sensation seeking among adolescents, and the latter may be an essential factor associated with injury. Future research should be conducted to further explore the relationship between sensation seeking and injuries.

The primary causes of injuries in this study were falls, hard objects, cuts or piercings, burns, transportation, and animal bites. A fall was defined as an event which resulted in a person coming to rest inadvertently on the ground or floor or other lower level [30]. Fall injuries are worth noting since many studies conducted in different populations have showed that falls were the most common causes of injuries [3,16]. Child falls may occur largely as a result of their innate curiosity of their surroundings, risk taking characteristics [30] and immature judgment. Adolescent falls may be associated with their personality like high-level activity. As suggested in the present study, gymnasiums of schools were the most-reported places where injuries occurred, and having PE class>2 classes/week was an independent risk factor for injury. Thus adolescent falls during sport activities in school, especially in the gymnasium, must not be overlooked. Additionally, the impact of fall injuries among students was great. Falls may result in sprains, dislocations, bruises, lacerations and cuts, and can even lead to disability and time off from school. Therefore, strategies targeting sports injuries, such as improving the environment of the gymnasiums in schools and strengthening the protection in sport activities are of great significance in adolescent injury prevention.

### Limitations and strengths

This study has several limitations. First, there were recall bias in the cross-sectional study since the self-reported method was used and the recall period of most information is in the past 12 months. In future research, a return visit will be conducted to check the information of participants to measure and control recall bias. Second, our research is an exploratory study, and the purposive sampling method is used. Third, the questionnaire could be improved, and some information was not collected, which might have caused underestimation of the effect of some factors or missing some other factors associated with injuries. Finally, the current study was conducted in Shenzhen City, making our findings difficult to generalize to other places. Future studies with better design among other populations are needed to confirm the findings of the study. Despite these limitations, we systematically analyzed characteristics and related factors for nonfatal injuries among adolescents and college students in Shenzhen city of China.

## Conclusions

In summary, nonfatal injuries have turned into a public health problem among adolescents and the college students in Shenzhen City, China. We found that the risk factors for injuries were male gender, self-perceived agrypnia, having PE class>2 classes/week, sexual behaviors, physical fighting, alcohol consumption, unsafe cycling, and skating in unsafe places. Falls were the leading cause of injuries and gymnasiums of schools were the most reported places where injuries occurred. Strategies targeting the above risk factors may be effective in the prevention of injuries. The strategies may include improving the environment of the gymnasiums in schools, strengthening protection in sport activities and encouraging safer behaviors related to cycling and swimming.

## Competing interests

The authors declared that they have no competing interests.

## Authors’ contributions

LZ participated in the design of the study and coordination, and helped draft the manuscript. DC participated in the analysis and interpretation of the data, and drafted the manuscript. GD participated in the design of the study, analysis of the data, and preparing the manuscript. All authors approved the final manuscript.

## Pre-publication history

The pre-publication history for this paper can be accessed here:

http://www.biomedcentral.com/1471-2458/13/392/prepub
